# A case report: autosomal recessive Myotonia congenita caused by a novel splice mutation (c.1401 + 1G > A) in *CLCN1* gene of a Chinese Han patient

**DOI:** 10.1186/s12883-018-1153-x

**Published:** 2018-09-22

**Authors:** Jing Miao, Xiao-jing Wei, Xue-mei Liu, Zhi-xia Kang, Yan-lu Gao, Xue-fan Yu

**Affiliations:** Department of Neurology and Neuroscience Center, the First Affiliated Hospital of Jilin University, Changchun, 130021 Jilin People’s Republic of China

**Keywords:** Myotonia congenita, Autosomal recessive, Case report, *CLCN1* gene

## Abstract

**Background:**

Autosomal recessive Myotonia congenita (Becker’s disease) is caused by mutations in the *CLCN1* gene. The condition is characterized by muscle stiffness during sustained muscle contraction and variable degree of muscle weakness that tends to improve with repeated contractions.

**Case presentation:**

A 21-year-old man presented with transient muscle stiffness since the last 10 years. He had difficulty in initiating movement and experienced muscle weakness after rest, which typically improved after repeated contraction (warm-up phenomenon). There was no significant family history. Medical examination showed generalized muscle hypertrophy. Serum creatine kinase level was 2-fold higher than the normal value. Electromyogram showed myotonic discharges. DNA sequence analysis identified a novel splice mutation (c.1401 + 1G > A) and a known mutation (c.1657A > T,p.Ile553Phe). He rapidly responded to treatment with mexiletine 100 mg three times a day for 6 months.

**Conclusions:**

This case report of autosomal recessive Myotonia congenita caused by a novel compound heterozygous mutation expands the genotypic spectrum of *CLCN1* gene.

## Background

Autosomal recessive Myotonia congenita (Becker’s disease) is caused by mutations in *CLCN1* gene, which is located on the 7q35 chromosomal region and contains 23 coding exons. The condition is characterized by muscle stiffness during sustained muscle contraction and a variable degree of muscle weakness, which tends to improve with repeated contraction. The *CLCN1* gene is responsible for chloride (Cl^−^) conductance in skeletal muscles, which contributes significantly to the repolarization of action potential.

Till date, several mutations of the CLCN1 gene such as missense and nonsense mutations have been reported in different populations across the world. To the best of our knowledge, only a few splicing mutations of the CLCN1 gene have been reported. These mutations may induce depolarisation of the skeletal muscle cell membrane which leads to hyperexcitablity (myotonia), which may improve on treatment with sodium (Na^+^) blocking agents such as mexiletine [1–5]. In this study, we report a Chinese patient suffering from recessive Myotonia congenita caused by compound heterozygous mutations which includes a novel splicing mutation.

## Case presentation

**Fig. 1:**
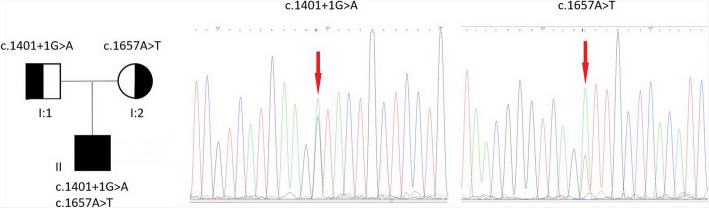
Pedigree and mutation analysis of the patient. The c.1401 + 1 G > A mutation (red arrow) and c.1657 A > T mutation (red arrow), inherited from his father (I:1) and mother (I:2) respectively, were found in the patient (II)

A 21-year-old man complained of transient muscle stiffness since 10 years. He experienced difficulty in initiating movement and felt muscle weakness after rest. However the symptoms typically improved after repeated contraction (warm-up phenomenon). The symptoms tended to aggravate during cold weather. He was unable to open his eyes immediately after washing his face with cold water. He had non-consanguineous parents and there was no significant family history. Further, the patient showed normal growth and development. Medical examination showed generalized muscle hypertrophy and normal muscle strength as assessed with the Medical Research Council (MRC) sum score. Deep tendon reflexes were attenuated. No nerve dysfunction or sensory deficit was noted. The serum creatine kinase level was 2-fold higher than the upper limit of the normal reference level. Electromyogram showed myotonic discharges. Biceps muscle biopsy specimen was obtained after written informed consent of the patient. The specimen was precooled with isopentane and frozen in liquid nitrogen. A section of muscle biopsy specimen was stained with hematoxylin-eosin (HE) and modified Gomori’s trichrome (MGT). Activity of oxidative enzymes such as succinate dehydrogenase (SDH), NADH-tetrazolium reductase (NADH-TR), and cytochrome c oxidase (COX) were normal. Next generation sequencing of the DNA sample of this patient identified a novel splice mutation (c.1401 + 1G > A) (which was inherited from his father) and a known mutation (c.1657A > T, p.Ile553Phe) (which was inherited from his mother) (Fig. 1). The novel splice mutation was not detected in Human Gene Mutation Database or any of the 200 healthy controls. The splicing site, analyzed by Human Splicing Finder, is implicated in the alteration of the wild-type donor site and most probably have an impact on splicing. Mutation Taster software predicted the effects of the mutation to be ‘disease causing’. The patient was prescribed mexiletine 100 mg three times per day for about 6 months. The patient responded well to the treatment and was completely relieved of his symptoms.

## Discussion and conclusions

Our patient showed transient muscle stiffness and weakness after rest which improved with repeated contractions. The symptoms were aggravated by cold weather and stress. EMG revealed myotonic discharges and gene sequencing analysis identified novel compound heterozygous mutations in *CLCN1* gene. Finally, a diagnosis of recessive myotonia congenita was established.

The *CLCN1* gene encodes the major skeletal muscle chloride channel CLCN-1. CLCN-1 plays an important part in the maintenance of resting potential, which is activated during the course of depolarization. The mutations of *CLCN1* gene were scattered throughout the entire sequence of the channel protein. The mutations included insertion/deletion, missense, nonsense, and splicing mutations. To the best of our knowledge, very few studies have reported splicing mutations of *CLCN1* gene [1, 2, 4, 5]. The sequences associated with splicing mutation contains two splice sites: one is a donor site, which is an invariant GU at the 5′ end of the intron and another is an acceptor site, which is an invariant AG at the 3′ end of the intron. Meyer-Kleine et al.*..* reported a 5′ splice-donor mutation (c.1471 + 1G > A) in three GM family, which can change the highly conserved consensus sequence [4]. Gianna et al reported four cases that harbored splicing mutations(c.563G > T, c.1169-5 T > G, c.1251 + 1G > A and c.1931-2A > G), which led to skipping exons in a frameshift change during RNA translation or premature termination [5]. In our case, the novel splicing mutation c.1401 + 1G > A in intron 12 affected the 5′ splice-donor sequences resulting in exons skipping and generation of out-of-frame mRNA. However, the specific mechanism is yet to be elucidated. Another mutation (c.1657A > T,p.Ile553Phe) has been reported in Chinese GM patients [6], which may induce small changes in channel properties. This indicates that the mutation may be associated with certain ethnic populations. CLCN-1 is a voltage-dependent ion channel that mediates chloride conductance in the skeletal muscle cell membrane. The mutations of CLCN1 gene may result in a decrease in channel opening probability and render the membrane hyperexcitable, which is manifested as myotonia. This can be improved by administration of Na^+^ blocking agents, such as mexiletine [7].

In conclusion, this case report of autosomal recessive myotonia congenita caused by a novel compound heterozygous mutation expands the genotypic spectrum of *CLCN1* gene.

## Abbreviations

COX: cytochrome c oxidase
GM: generalized myotonia
HE: hematoxylin-eosin
MGT: modified Gomori’s trichrome
MRC: Medical Research Council
NADH-TR: NADH-tetrazolium reductase
SDH: succinate dehydrogenase
